# A class of Fourier integrals based on the electric potential of an elongated dipole

**DOI:** 10.1186/2193-1801-3-729

**Published:** 2014-12-12

**Authors:** Georgios Aim Skianis

**Affiliations:** Department of Geography and Climatology, Faculty of Geology and Geo-Environment, University of Athens, Panepistimiopolis, Athens 157 84 Greece

**Keywords:** Fourier integral, Electric dipole, Electric potential, Polarization, Spatial frequency, Green’s function

## Abstract

In the present paper the closed expressions of a class of non tabulated Fourier integrals are derived. These integrals are associated with a group of functions at space domain, which represent the electric potential of a distribution of elongated dipoles which are perpendicular to a flat surface. It is shown that the Fourier integrals are produced by the Fourier transform of the Green’s function of the potential of the dipole distribution, times a definite integral in which the distribution of the polarization is involved. Therefore the form of this distribution controls the expression of the Fourier integral. Introducing various dipole distributions, the respective Fourier integrals are derived. These integrals may be useful in the quantitative interpretation of electric potential anomalies produced by elongated dipole distributions, at spatial frequency domain.

## 1. Introduction

**Figure 1:**
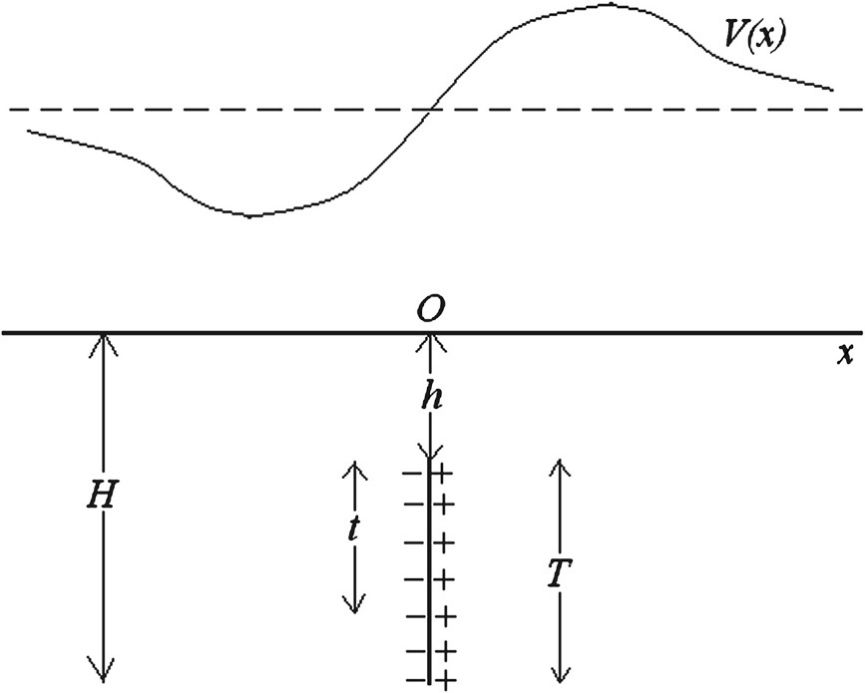
**A representation of the electric field which is produced by a vertical fault.**

In earth sciences it is well known that the electric field which is produced by a fault with a geothermal activity may be simulated by a system of dipoles oriented perpendicularly to the surface of the fault (Corwin and Hoover 1979, Fitterman 1984). The geometry of the geothermal field is presented in Figure 1. A vertical fault with an infinite horizontal dimension is considered. *h* is the depth of the roof of the fault. *T* is its vertical dimension. *t* is the distance of a certain dipole from the roof of the fault and it takes values between 0 and *T. x* is the location of a point at ground surface. The Green’s function of the electric potential *V*(*x*) is the potential of a dipole with a small length and polarization *m*(*t*) per unit length *t* (Fitterman 1979). *m*(*t*) may be constant with depth, but it may also increase with *t* (Corwin and Hoover 1979, Corwin et al. 1981).

If the dipole has an infinite horizontal dimension and a dipole axis parallel to ground surface, it produces an elementary electric potential *dV*(*x*), which is given by (Murty and Haricharan 1985):
1

The electric potential *V*(*x*) is given by:
2

The quantitative interpretation of the electric potential *V*(*x*) may be done at space domain, according to equation (2). It can also be done at spatial frequency domain, by taking the Fourier transform *U*(*u*) of *V*(*x*), according to the relation:
3

*u* is the spatial frequency.

For various expressions of *m*(*t*) a closed form of the integral *V*(*x*) can not be easily found. On the other hand, it is possible to derive the Fourier transform *U*(*u*) in a closed form, for certain types of the function *m*(*t*). In geophysics, the quantitative interpretation of potential anomalies in spatial frequency domain is quite usual and may give reliable results (Odegard and Berg 1965, Rao et al. 1982, Skianis et al. 2006, Skianis 2012). Therefore the derivation of expressions of *U*(*u*) may have applications in geosciences and, possibly, in other fields of physics and engineering, as concerns the behaviour of the electrical field produced by a dipole distribution.

The subject of the present paper is the derivation of non tabulated expressions of the Fourier integral of *V*(*x*) of equation (2), for various forms of *m*(*t*). An appropriate change of the order of integration must be done before proceeding to the derivation of the integral transform. Consequently, various integrals can be obtained, in closed form, for different expressions of *m*(*t*).

## 2. A general form for the Fourier integral *U*(*u*)

In the following mathematical analysis, it is assumed that *u* > 0. For *u* = 0, *U*(0) = 0, since the function *V*(*x*) is antisymmetric, as it can be seen from equations (2), (3) and from Figure 1. On the other hand, for *u* < 0 the Fourier integral *U*(*u*) is the complex conjugate of *U*(|*u*|). Therefore, knowledge of *U*(*u*) for *u* ≥ 0 is sufficient to describe the behaviour of the Fourier integral at negative *u* values.

Combining equations (2) and (3) and changing the order of integration, *U*(*u*) takes the form:
4

It is well known (Spiegel 1976) that:
5

Plugging equation (5) into (4) gives:
6

Therefore, the Fourier transform of *V*(*x*) may be expressed as the product of the function -*iπ*exp(-*hu*) times the integral of *m*(*t*)exp(-*ut*) for *dt*. Equation (6) may be used in deriving the Fourier integral *U*(*u*) for various forms of *m*(*t*).

## 3. The Fourier integral *U*(*u*) for a constant *m*(*t*)

For *m*(*t*) = 1 equation (6) becomes:
7

It can be easily found that:
8

Further, for *m*(*t*) = 1 the equation (2) for *V*(*x*) becomes:
9

In order to derive a closed expression for *V*(*x*) the following tabulated integral (Spiegel 1976) has to be taken into account:
10

Putting *z* = *t* + *h* and substituting in equation (10), the closed expression for *V*(*x*) in equation (9) can be found. *V*(*x*) is given by:
11

Therefore, according to the equations (8) and (11) the following transform pair *FT* is obtained:
12

## 3. The Fourier integral for *m*(*t*) proportional to *t*

For *m*(*t*) = *t* equation (6) becomes:
13

It can be easily found that:
14

On the other hand, since *m*(*t*) = *t*, the equation (2) for *V*(*x*) becomes:
15

Taking into account the tabulated integral of equation (10) and making a proper change of variable (*z* = *t* + *h*), it can be found, after some algebraic manipulation, that:
16

According to the equations (14) and (16) the following Fourier transform pair is obtained:
17

In case that *h* = 0, which physically means that the roof of the geothermal fault is located at ground surface, equation (16) becomes:
18

Equation (14) becomes:
19

According to the equations (18) and (19) the following Fourier transform pair is obtained:
20

## 4. The Fourier integral for *m*(*t*) varying exponentially with *t*

In the previous paragraphs, *m*(*t*) had such a form that a closed expression for *V*(*x*) could be found, according to the equation (2). Furthermore, the Fourier transform pairs of equations (12), (17) and (20), could be derived straight from equation (3), by integrating for *x* and taking into account tabulated Fourier integrals which may be found in Spiegel (1976) and Abramowitz and Stegun (1968). There are cases, however, that *m*(*t*) has such a form that the closed expression for *V*(*x*) can not be found by tabulated integrals, therefore a closed expression for *U*(*u*) can not be derived by equation (3). This happens, for example, when an exponential function exp(*at*), with *a* real is involved in the expression for *m*(*t*). In such a case, equation (6) may be the only way to find *U*(*u*) in a closed form.

An alternative approach would be to expand exp(*at*) to a series of (*at*)^*n*^/*n*!, express equation (2) as a sum of definite integrals ((*at*)^*n*^/*n*!)d*t*/(*x*^2^ + (*t* + *h*)^2^), find the Fourier integral of each separate term according to equation (3) and try to find a closed expression for the sum of infinite terms. The whole procedure seems quite tedious and it is not sure if a closed expression for *U*(*u*) may be derived. Therefore, it is more convenient to proceed according to equation (6).

For *m*(*t*) = exp(*at*) the expression for *V*(*x*), according to the equation (2), is:
21

There are no tabulated integrals to find a closed expression for *V*(*x*), according to the equation (21). It is possible, however, to find the Fourier integral of *V*(*x*). For *m*(*t*) = exp(*at*) the equation (6) becomes:
22

It can be easily found that:
23

Taking into account that *u* is positive, the denominator at the right part of equation (23) becomes zero at *u* = *a* for *a* > 0. It can be easily proved, however, that *U*(*u*) is continuous at this point and that:
24

According to the equations (3), (21) and (23), the following Fourier transform pair is obtained:
25

## 4. Some Fourier integrals for an infinitely big *T*

The case of an infinitely big *T* represents a fault with a very big vertical dimension. The function *V*(*x*), according to the equation (2), may be defined if polarization *m* is zero for *t* equal to zero. Three forms of the function *m*(*t*) are considered.

In the first case, *m*(*t*) is given by:
26

with *b* > 0.

**Figure 2:**
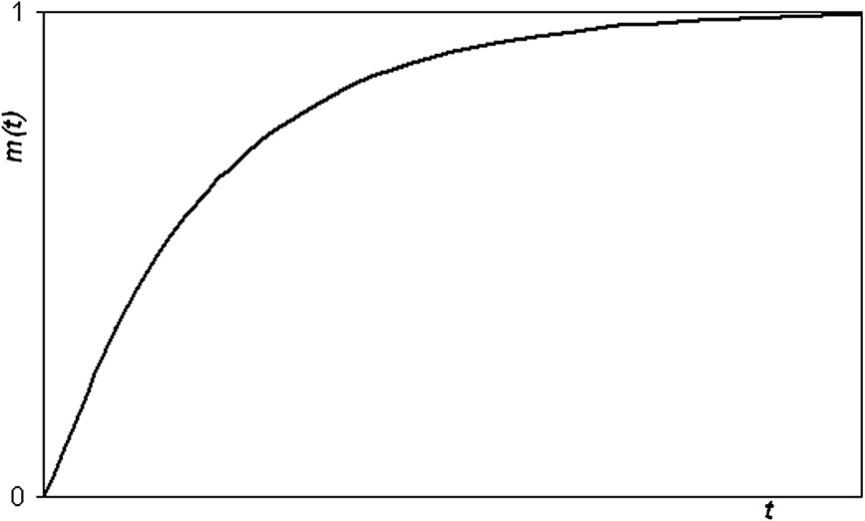
**Variation of the polarization** ***m*** **against vertical dipole distance** ***t*** **from the roof of the fault.**

A polarization *m*(*t*) of the type of equation (26) ensures that the potential *V*(*x*) takes finite values. From the physical point of view it expresses a fault of geothermal activity with an increasing polarization with depth, which tends to a constant value. In Figure 2 the variation of *m* with *t* is presented.

Equation (2) becomes:
27

Combining the equations (3), (6) and (27) gives:
28

It can be easily found that:
29

Combining the equations (3), (27) and (29), the following Fourier transform pair is obtained:
30

The function *m*(*t*) = 1-exp(-*bt*) is equal to zero for *t* = 0 and increases with *t* tending to unity.

The function *m*(*t*) = *erf*[sqrt(*bt*)] (*erf* is the error function) has a similar behaviour with *t*, since it is zero for *t* = 0 and tends to unity as long as *t* increases. It also ensures finite values of *V*(*x*). Taking into account the equation (2), the expression for *V*(*x*) becomes:
31

Combining the equations (3), (6) and (31) gives:
32

It is well known (Spiegel 1976, Abramowitz and Stegun 1968) that:
33

Combining the equations (3), (31), (32) and (33), the following Fourier transform pair is obtained:
34

The third case is that of *m*(*t*) = *t*^*n*^, for 0 < *n* < 2.

According to the equation (2), the expression for *V*(*x*) becomes:
35

Combining the equations (3), (6) and (35) gives:
36

It is well known (Abramowitz and Stegun 1968) that:
37

Γ is the Gamma function.

Combining the equations (3), (35), (36) and (37) the following Fourier transform pair is obtained:
38

Equation (38) is valid for 0 < *n* < 2. For *n* greater than or equal to 2 the generalised integral of equation (2) does not converge to a finite number.

It is important to mention that the generalized integrals on the right side of equations (28), (32) and (36) are the Laplace transforms of the respective polarization functions *m*(*t*).

## 5. Conclusions

A class of non tabulated Fourier transform pairs have been derived, based on the Green’s function of the potential of a distribution of elongated electric dipoles. The Fourier integrals may be expressed as the product of the Fourier transform of the Green’s function exp(*-hu*) times an integral which depends on the polarization function *m*(*t*). For an infinite vertical dimension *T*, this integral is actually the Laplace transform of *m*(*t*).

The Fourier integrals are expressed in rather simple closed forms and they can be used in the direct or iterative quantitative interpretation of surface electric potential measurements at geothermal fields (Corwin and Hoover 1979, Corwin et al. 1981, Thanassoulas and Lazou 1993, Apostolopoulos et al. 1997, Jouniaux and Ishido 2012). Further applications may possibly be developed in modelling of electric fields produced by distributions of elongated electric dipoles.
